# Empirical support for a multi-dimensional model of sensations experienced by youth during their initial smoking episodes

**DOI:** 10.1111/j.1360-0443.2010.03041.x

**Published:** 2010-10

**Authors:** Chris G Richardson, Chizimuzo T C Okoli, Pamela A Ratner, Joy L Johnson

**Affiliations:** 1 School of Population and Public Health, University of British ColumbiaVancouver, Canada; 2 IMPART, University of British ColumbiaVancouver, Canada; 3 School of Nursing, University of British ColumbiaVancouver, Canada

**Keywords:** adolescent, factor analysis, initial sensations, tobacco smoking

## Abstract

**Aims:**

To examine the dimensionality of sensations experienced during initial tobacco smoking.

**Design:**

Cross-sectional survey.

**Setting:**

Thirteen secondary schools located in British Columbia, Canada.

**Participants:**

Data from 1187 adolescents who responded ‘yes’ to the question: ‘Have you ever tried cigarette smoking, even one or two puffs?’.

**Measurements:**

Participants answered questions about their demographic characteristics, tobacco smoking history and sensations experienced during their initial smoking episodes.

**Findings:**

The sensations appear to represent the following three separate but modestly correlated dimensions: a pleasant dimension defined by feeling good and relaxed; an unpleasant dimension defined by coughing, feeling sick and nervous; and a ‘buzz’ dimension defined by feeling high and dizzy. The three factors made statistically significant contributions to the prediction of transition to regular smoking (defined as having smoked at least 100 cigarettes in one's life-time) after adjusting for age, sex and age at first puff.

**Conclusions:**

The results suggest that three relatively distinct physiological systems appear to explain the relationship between initial smoking sensations and probability of becoming a regular smoker. Researchers examining sensations experienced during initial tobacco smoking episodes should consider using a three-dimensional profile of symptoms composed of pleasant, unpleasant and buzz dimensions.

## INTRODUCTION

Although many risk factors for developing tobacco dependence are likely to be present before the onset of tobacco exposure, some can only be observed after exposure to tobacco (or nicotine) [1]. The nature of sensations (e.g. nausea, coughing, dizziness, relaxation and light-headedness) experienced during initial episodes of smoking represents one such set of risk factors. Individual differences in these sensations have been identified as an important predictor of the transition from experimental to more regular smoking and to the development of tobacco dependence [2–6].

Researchers speculated primarily that the experience of unpleasant sensations during initial smoking episodes deterred future smoking [7] by discouraging further experimentation with tobacco, and in so doing prevented the development of tolerance and dependence [8]. Individual differences in the experience of aversive sensations are thought to be the result of variation in the extent to which nicotine consumption in the form of smoked tobacco triggers negative sensations via stimulation of the digestive system (e.g. nausea) and coughing [9].

In addition to the deterrent effects of aversive symptoms, researchers have reported that experiencing pleasant sensations (e.g. feeling relaxed) during initial smoking episodes reinforces subsequent smoking behavior [3]. Individual differences in the experience of pleasant or euphoric sensations are thought to be the result of differences in the extent to which nicotine stimulates the release of neurotransmitters (e.g. dopamine) associated with the brain's reward system [10,11].

More recently, researchers have also proposed a general sensitivity hypothesis in which the experience of any initial sensation, pleasant or unpleasant, reflects an underlying sensitivity to nicotine [4,6,8]. Guided by non-human research examining the influence of genetics, and other factors, on nicotine sensitivity and patterns of reinforcement [12,13], this sensitivity has been interpreted as an indicator of vulnerability to nicotine dependence characterized by a more rapid development of tolerance and more intense self-administration of nicotine [6]. Support for this hypothesis has come from evidence suggesting that genetic polymorphisms related to D4 and D2 receptors appear to modulate initial sensitivity to nicotine [14].

Researchers investigating the relationship between tobacco dependence and initial sensations typically compare the retrospective self-reports of the initial smoking experiences of current smokers with those of non-smokers (for a recent exception see Acosta *et al*. [15]). The relationships between the retrospectively reported sensations and current smoking status have been examined for individual sensations and by intuitively combining sensations into pleasant or unpleasant scores. For example, researchers have found that youths who report experiencing positive sensations are more likely to develop regular smoking patterns in the future [3,16], whereas youths who report more unpleasant sensations are less likely to progress towards regular smoking [5].

Although the practice of organizing symptoms into pleasant and unpleasant groups is widespread, evidence supporting the general sensitivity hypothesis suggests that this classification may not be appropriate. For example, researchers have found that irrespective of whether sensations were designated as pleasant or unpleasant, the number of sensations experienced during an initial smoking episode was associated significantly with an increased risk of smoking [17]. However, they also found that specific sensations, such as having noticed the smell of a burning cigarette, and having experienced a buzz, rush, relaxation or dizziness, were associated with current smoking, but having experienced nausea was associated negatively with current smoking [17]. In contrast, others have found that youths' reports of nausea, dizziness or relaxation predicted transition to tobacco dependence positively, whereas those who experienced irritation were less likely to have become regular smokers [4]. Furthermore, a study of adult women found that, although reports of having ‘pleasurable sensations’ (buzz and relaxation) during the initial smoking episode were associated positively with having become a current smoker, reports of ‘unpleasant sensations’ (nausea and cough) did not differ between smokers and non-smokers [3].

One possible reason for these contradictory results is that a particular researcher-imposed classification or dimensionality (e.g. intuitively grouping ‘pleasant’ and ‘unpleasant sensations’) may not represent the true relationships among the different sensations. If the grouping of sensations does not reflect the underlying dimensionality of the construct(s) under investigation, then subsequent analyses involving ‘sensation group’ scores will be confounded by measurement error. To test the hypothesis that the sensations experienced during initial tobacco smoking episodes represent multiple dimensions, we examined the dimensionality of a commonly described set of sensations experienced during an initial smoking episode using a combination of exploratory and confirmatory factor analyses.

## METHODS

### Setting and participants

The analyses presented in this study are based upon data from 1187 adolescents who responded ‘yes’ to the question: ‘Have you ever tried cigarette smoking, even one or two puffs?’ in the British Columbia Youth Survey on Smoking and Health (BCYSOSH 1). The BCYSOSH 1 was a cross-sectional survey administered in 2001 to a sample of 3280 high school students in British Columbia, Canada. More detailed information about the sampling strategy and survey design can be found in earlier publications [18,19]. See Table 1 for participant characteristics.

**Table 1:** Participant demographics and smoking-related characteristics

Participant characteristics (n = 1187)	
Percentage of female participants	51
Average age in years (standard deviation)	16 (0.9)
Average age at first puff in years (standard deviation)	12 (2.7)
Reported life-time number of cigarettes smoked	
Only a few puffs (%)	439 (37)
1–5 cigarettes (%)	221 (19)
6–15 cigarettes (about half a pack in total) (%)	82 (7)
16–25 cigarettes (about one pack in total) (%)	83 (7)
26–99 cigarettes (less than five packs in total) (%)	95 (8)
More than 100 cigarettes (more than five packs in total) (%)	256 (22)
Sensations experienced at time of initial smoking episode	
Felt dizzy (%)	434 (37)
Coughed (%)	738 (62)
Felt sick (%)	287 (24)
Felt high (%)	222 (19)
Felt relaxed (%)	456 (38)
Felt nervous (%)	290 (24)
Felt good (%)	356 (30)

### Measures

#### Demographic variables

Demographic variables included in the analyses were participants' age (in years) and sex (male or female).

#### Ever tried cigarette smoking

The participants were asked, ‘Have you ever tried cigarette smoking, even one or two puffs?’. Response options were ‘yes’ or ‘no’.

#### Age of smoking initiation

The participants were asked: ‘How old were you when you took your first puff of a cigarette?’. Respondents were asked to provide their age at first puff in years.

#### Transition to regular smoking

Participants were asked: ‘About how many cigarettes have you smoked in your entire life?’, with the following response options: ‘None’, ‘I have only had a puff or puffs of a cigarette’, ‘one to five cigarettes’, ‘six to 15 (about half a pack total)’, ‘16–25 (about one pack total)’, ‘26–99 (fewer than five packs total)’ and ‘more than 100 (more than five packs total)’. Responses to this question were dichotomized to either fewer than 100 (no transition) or more than 100 cigarettes (transition to regular smoking) [20].

#### Sensations experienced at time of initial smoking episode

The participants were asked: ‘When you first started to smoke cigarettes did you: feel dizzy, cough, feel sick, feel high, feel relaxed, feel nervous or feel good?’. Each of the sensations was listed on a separate line and the response options were ‘yes’, ‘no’ or ‘I have never smoked’. These possible sensations were derived from the literature [2,3].

#### Current smoking status

To assess current smoking status, participants were asked: ‘Have you smoked at least once in the past month?’. Participants responding ‘yes’ were coded as 1 and those responding ‘no’ were coded as 0.

### Data analysis

The analyses in this investigation were conducted in four stages.

#### Stage 1: creating exploratory factor analysis (for EFA) and confirmatory factor analysis (for CFA) sample groups

To ensure that the exploratory and confirmatory sample groups contained an equal number of current smokers, participants were stratified initially into current smokers and non-smokers based on whether they had smoked at least once in the past 30 days. Members of each smoking group were then assigned randomly to either the exploratory sample or the confirmatory sample.

#### Stage 2: EFA with the exploratory sample

To accommodate the binary nature of the sensations and potentially correlated factors, robust EFA (unweighted least-squares estimation using tetrachoric correlations with PROMAX rotation) was used to produce solutions composed of one, two and three factors using the software Mplus version 4 [21]. To evaluate the appropriateness of the factor solutions we examined the interpretability of the factor solutions, scree plots, number of eigenvalues greater than 1.0 and the root mean square error of approximation (RMSEA) values.

#### Stage 3: evaluating model fit using CFA with the confirmatory sample

Using the software Mplus version 4, we compared the fit of the one-factor, two-factor and three-factor models, derived in stage 2, using CFA. To accommodate the binary nature of the data, we used mean and variance adjusted weighted least-squares estimation [22].

#### Stage 4: predicting transition to regular smoking using the full sample

Multivariate logistic regression models were estimated to examine the extent to which each of the latent factors of the best-fitting model (identified in stages 2 and 3) contributed to the prediction of the adolescents' transition to regular smoking (defined as having smoked at least 100 cigarettes in one's life-time). To facilitate interpretation of the results, the primary analyses for this stage were performed by creating dichotomous scores on each factor using the following criteria: ‘0’ if they did not experience any factor-specific symptoms and ‘1’ if they experienced one or more factor-specific sensations. As a precaution, a parallel set of secondary analyses was conducted that used saved factor scores from CFA models (run on the full sample) as continuous scores of the factors in the multiple regression models predicting transition to regular smoking.

## RESULTS

### 

#### Stage 1: creating exploratory and confirmatory groups

The exploratory sample contained 593 respondents (average age of 16 years; 48% female; 34% reported smoking at least once in the previous 30 days; and 22% reported smoking at least 100 cigarettes in their life-time). The confirmatory sample contained 594 respondents (average age of 16 years; 53% female; 34% reported smoking at least once in the previous 30 days; and 22% reported smoking at least 100 cigarettes in their life-time).

#### Stage 2: EFA with the exploratory sample

The scree plot (major point of inflection between two and three factors) and the number of eigenvalues greater than 1.0 both supported a two-factor solution. The standardized factor loadings from the exploratory factor analyses of the exploratory data for one-, two- and three-factor solutions are presented in Table 2.

**Table 2:** Estimated factor loadings for one-, two- and three-factor exploratory factor analysis (EFA) models

(n = 593)	One-factor model	Two-factor model		Three-factor model		
			
Sensation	Sensitivity	Pleasant	Unpleasant	Pleasant	Unpleasant	Buzz
Cough	0.27	−0.16	0.52	0.10	0.69	−0.18
Sick	0.15	0.05	0.82	−0.16	0.66	0.29
Nervous	0.17	−0.04	0.50	0.07	0.57	−0.03
Dizzy	−0.46	0.65	0.35	0.06	0.15	0.70
High	−0.59	0.74	0.21	0.06	−0.03	0.81
Relaxed	−0.88	0.79	−0.35	0.73	−0.14	0.15
Good	−0.94	0.85	−0.31	0.97	0.00	0.06
RMSEA	0.20	0.05		0.01		

RMSEA: root mean square error of approximation.

The one-factor solution representing a general measure of sensitivity appeared to represent primarily pleasant sensations (i.e. feeling good and relaxed), with substantially smaller loadings (in the opposite direction) for the unpleasant items (cough, sick, nervous). The RMSEA (0.20) for this solution suggested a poor fit to the data [23]. The RMSEA of the two-factor solution was substantially smaller (i.e. better-fitting) than the one-factor solution's and indicated relatively good fit to the data. The first factor of the two-factor solution appeared to represent a pleasant dimension with the dizzy item also loading positively on the second factor and relaxed item loading in the opposite direction on the second factor. The second factor of the two-factor solution appeared to represent an unpleasant dimension defined primarily by feeling sick. Although the two factors were not correlated (*r* < 0.01), there were relatively large cross-loadings (0.35) for two of the items (i.e. dizzy and relaxed). The RMSEA of the three-factor solution suggested an even better fit to the data and all the items loaded primarily on only one of the factors (i.e. all secondary loadings were less than 0.30). The first factor in this solution was labelled a pleasant dimension (i.e. feeling good and relaxed); the second factor was labelled an unpleasant dimension defined primarily by coughing and feeling sick; and the third factor, labelled a ‘buzz’ dimension, was defined by feeling high and dizzy. The buzz dimension was correlated positively with the pleasant dimension (*r* = 0.51) and there was a negative correlation between the pleasant and unpleasant dimensions (*r* = –0.36).

#### Stage 3: evaluating model fit using CFA with the confirmatory sample

The results of the one-factor, two-factor and three-factor CFA models, conducted on data from the confirmatory sample, are presented in Table 3. Overall, the results of the CFA confirm the results of the previous EFA. The indicators of approximate fit all suggest strongly that the three-factor solution is the best-fitting solution (i.e. the CFI was highest in the three-factor model and the RMSEA and SRMR were lowest in the three-factor model). Additionally, the three-factor model came close to reaching the criteria for good fit (CFI ≥ 0.95, RMSEA ≤ 0.06, and SRMR ≤ 0.08) recommended by Hu & Bentler [24].

**Table 3:** Standardized loadings of the confirmatory factor analyses (CFA) of the one-, two- and three-factor models

(n = 594)	One-factor model	Two-factor model		Three-factor model		
			
Item	Sensitivity	Pleasant	Unpleasant	Pleasant	Unpleasant	Buzz
Cough	−0.21		0.56		0.37	
Sick	−0.04		0.63		10.00	
Nervous	−0.19		0.61		0.44	
Dizzy	0.40	0.39				0.70
High	0.58	0.58				0.80
Relaxed	0.85	0.85		0.84		
Good	0.95	0.96		0.98		
Model fit						
χ^2^ (df)	216 (10)	154 (10)		47 (8)		
CFI	0.74	0.82		0.95		
RMSEA	0.18	0.16		0.09		
SRMR	0.18	0.14		0.09		

CFI: Comparative Fit Index; RMSEA: root mean square error of approximation; SRMR: standardized root mean residual.

The inter-factor correlations from the CFA of the three-factor solution indicated that the buzz dimension was associated positively with both the pleasant dimension (0.51) and the unpleasant dimension (0.38) and that there was a small negative correlation between the pleasant and unpleasant factors in both the two-factor (–0.16) and three-factor solutions (–0.26).

#### Stage 4: predicting transition to regular smoking using the full sample

Because the factors in the three-factor model were interpretable and provided the best fit to the data, a series of logistic regression models were used to examine the extent to which each of the three sensation factors contributed to the prediction of transition to regular smoking (defined as having smoked at least 100 cigarettes in one's life-time) after controlling for age, sex and age at first puff. Each of the sensation factors was scored using the following criteria: ‘0’ if they did not experience any factor-specific symptoms and ‘1’ if they experienced one or more factor-specific sensations. We also included all possible two-way interactions between sex and the sensation factors and between each potential pair of sensation factors in the initial model (results not shown). However, the only interaction that was significant in these models was the interaction involving the buzz and pleasant factors.

The results of the final model containing the single interaction term are reported in the top portion of Table 4. As the participants' age increased so did their odds of having smoked 100 or more cigarettes, and as age at first puff increased, the odds of smoking 100 or more cigarettes decreased significantly. Participants experiencing at least one unpleasant sensation were 55% less likely to have smoked 100 or more cigarettes compared with those who had not experienced one or more unpleasant sensations. Although both the buzz and pleasant factors were significant predictors in the model, there was a significant interaction between these two factors. In the context of this model, the odds ratio (OR) for the interaction term can be interpreted as a ratio of predicted odds ratios for the main effects [25,26]. More specifically, the OR [95% confidence interval (CI)] for buzz was 10.95 (5.90, 20.10) when no pleasant sensations were reported and 3.69 (2.40, 5.68) when one or more pleasant sensations were reported. Additionally, the OR (95% CI) associated with the experience of one or more pleasant sensations was 7.75 (4.15, 14.50) when no buzz sensations were reported and 2.61 (1.71, 3.99) when one or more buzz sensations were reported.

**Table 4:** Results of multiple logistic regression models predicting life-time cigarette use

		95% CI for odds ratio	
		
(n = 1164)	Odds ratio	Lower	Upper
Model using dichotomous scores for factors			
Age (years)	1.68*	1.41	1.99
Age at first puff (years)	0.82*	0.77	0.87
Sex	1.18	0.85	1.64
Unpleasant	0.45*	0.31	0.65
Buzz	10.95*	5.94	20.20
Pleasant	7.75*	4.15	14.47
Buzz × pleasant interaction	0.34*	0.16	0.71
Model using mean-centered saved factor scores			
Age (years)	1.68*	1.42	1.99
Age at first puff (years)	0.83*	0.78	0.88
Sex	1.09	0.78	1.51
Unpleasant	0.40*	0.23	0.68
Buzz	7.03*	3.76	13.16
Pleasant	2.39*	1.30	4.38
Buzz × pleasant interaction	0.26*	0.14	0.49

Response variable: smoked at least 100 cigarettes (yes or no). Model fit for dichotomous factor scores: *R*^2^ = 0.23 (Cox & Snell), 0.36 (Nagelkerke). Model χ^2^ (7 df) = 311, *P* < 0.001. Model fit for continuous mean-centered factor scores: *R*^2^ = 0.22 (Cox & Snell), 0.34 (Nagelkerke). Model χ^2^ (7 df) = 294, *P* < 0.001.

* *P* < 0.01.

#### Secondary analyses using factor scores

To generate factor scores for the latent factors, the three-factor CFA from phase 3 was run on the entire sample and the factor scores for each participant were saved. This model provided a relatively good fit to the data (χ^2^_(df = 8)_ = 63.9, CFI ≥ 0.97, RMSEA ≤ 0.08 and SRMR ≤ 0.07). After mean centering the saved factor scores, a series of logistic regression models were then used to examine the extent to which each of the three sensation factor scores made independent contributions to the prediction of transition to regular smoking after controlling for age, sex and age at first puff. All possible two-way interactions between sex and the three sensation factors and between all possible pairs of the sensation factors were included in the initial model (results not shown). However, the only interaction that was significant in this model was the interaction involving the buzz and pleasant factors. The results of the final model of this secondary analysis, with the single interaction term, were very similar to the analyses carried out using dichotomized factor scores and are reported in the bottom portion of Table 4.

## DISCUSSION

We conducted a series of factor analytical tests to examine the dimensionality of sensations experienced by adolescents during their initial smoking episodes. Exploratory factor analyses (EFA) and confirmatory factor analyses (CFA) indicated that seven sensations experienced by adolescents during their initial smoking episodes appear to represent three separate but modestly correlated factors. The first factor was interpreted as representing a pleasant dimension (i.e. feeling good and relaxed); the second factor appeared to represent an unpleasant dimension defined primarily by coughing, feeling sick and nervous; and the third factor, defined by feeling high and dizzy, was interpreted as a ‘buzz’ dimension.

The presence of separate pleasant and unpleasant dimensions was expected, given their extensive mention in the literature and similar findings in other published studies [15,27]. Evidence of a separate buzz dimension characterized by feeling dizzy and high represents a new finding that might explain the lack of simple structure (i.e. EFA items loading primarily on one factor) cited in the literature examining the dimensionality of sensations. For example, researchers have found that the dizzy sensation had significant factor loadings on both pleasant and unpleasant factors [27]. Additionally, feeling dizzy has been reported to have a relatively strong loading on both the pleasant sensation factor (0.50) and on the unpleasant sensation factor (0.60) [15]. Although Acosta *et al*.'s factor analysis also produced a three-factor solution, their third factor was composed of items related to sensory/peripheral factors (e.g. taste, smell and feel in fingers); they did not report a four-factor solution [15] so we cannot determine whether the dizzy item would have formed a separate buzz factor akin to that found here.

From a psychometric perspective, the results of this study combined with similar findings of a strong cross-loading for the dizzy item [15,27] suggest that including responses to the dizzy item in the total score of either a pleasant or unpleasant factor will add considerable measurement error to any subsequent analyses involving summary scores. One solution to this problem is not to include the dizzy item in the calculation of pleasant or unpleasant factors, as was conducted by Rodriguez & Audrain-McGovern [27]. However, this approach results in a loss of potentially useful information. Our results suggest that the dizzy sensation should be treated as an indicator of a third factor that we characterized as feeling ‘buzzed’.

The use of a three-factor solution is supported further by our finding that the three factors made statistically significant contributions to the prediction of transition to regular smoking. We also found evidence indicating that the experience of ‘buzz’ sensations was associated with a substantial increase in the odds of progressing to smoking more than 100 cigarettes by youths who did not report any pleasant sensations (compared with those who reported at least one pleasant sensation). A very similar finding was reported by researchers who found that retrospective reports of light-headedness (i.e. feeling buzzed) experienced during initial cigarette smoking was related to having smoked at least 100 cigarettes only among respondents who also reported not liking their early smoking experiences [28]. These findings provide some support for the sensitivity hypothesis in that feeling buzzed was associated with increased odds of transitioning to regular smoking, particularly when respondents did not experience any of the pleasant sensations.

A possible explanation for the interaction between the buzz and pleasant factors is that youths' interpretations of their initial sensations may be influenced by the presence or absence of more experienced tobacco users. In their review of the literature about initial tobacco use episodes, Eissenberg & Balster [5] suggested that more experienced users of tobacco might minimize negative effects (e.g. nausea) while identifying other effects (e.g. dizziness) with positive descriptors, such as rush or buzz. In the context of our study, a portion of adolescents who did not report feeling dizzy may have actually felt dizzy but interpreted the sensation as feeling good. The possibility of a context-dependent interpretation of the dizzy sensation warrants further investigation, and reinforces our recommendation that it not be included as an indicator or measure of unpleasant sensations.

We also found that experiencing an unpleasant sensation was associated with a significant reduction in the odds of going on to smoke 100 or more cigarettes. This contradicts recent research suggesting that unpleasant sensations do not predict progression to nicotine dependence [16]. A potential explanation for this finding is that the experience of unpleasant sensations reflects an increased sensitivity to nicotine-induced dysphoric effects (e.g. nausea) [5] that is not as amenable to the effects of tolerance as those with decreased sensitivity to nausea [29]. Further support for the presence of a protective effect specific to the experience of unpleasant symptoms can also be found in recent investigations of the influence of specific genes on initial sensitivity to nicotine in non-smokers. In this research, Perkins *et al*. found that the presence of the DRD4 7 allele is associated with greater aversive responses to nicotine but does not predict pleasant symptoms (e.g. nicotine liking) [14].

Although the results of this study support a three-factor model of sensations experienced by adolescents during their initial smoking episodes, there are several limitations to be considered. As with all retrospective self-reported data, it is possible that recall bias may have influenced the results. However, our respondents were all adolescents and thus had experienced their initial sensations in a relatively recent time-frame, which reduces the potential for selective recall. Support for the validity of retrospective reports of early experiences with smoking has been provided by a study that simulated initial smoking experiences by exposing regular smokers to nicotine after they abstained from smoking for 5 days [30]. Additionally, the results of our factor analyses, particularly the cross-loading of the dizzy item, were very similar to the results found in a prospective study [15] that was not subject to recall bias.

Another limitation of this study involves our decision not to explore sex differences in our factor analyses. Although we did not find evidence of interactions between sex and sensation factors in our prediction of transition to regular smoking, recent research suggests that genetic polymorphisms related to dopamine D4 and D2 receptors may modulate some aspects of initial sensitivity to nicotine before the onset of nicotine dependence differentially between men and women [14]. We encourage further research about the impact of sex on the sensations experienced during initial episodes of smoking.

Another limitation to consider is that our analyses involved a pool of only seven sensations. Although these are among the most common sensations discussed in the literature, the inclusion of additional items in subsequent analyses may lead to the discovery of new factors or alternative interpretations of the factors identified in this study. For example, researchers reported recently that sensations of feeling high and feeling a rush tend to be classified as pleasant and somewhat pleasant, respectively, while the sensation of buzz tends to be perceived as unpleasant [31]. Although we interpreted feeling dizzy and high as indicators of a general buzz factor, our study did not include assessments of sensations characterized by the terms ‘buzz’ and ‘rush’. Finally, it is also possible that unaccounted factors, including other drug use such as alcohol or cannabis, may have influenced the initial sensations [32].

Given the results of this study and that of similar analyses assessing the dimensionality of initial sensations [15,27], we believe that there is compelling evidence to examine the sensations experienced during initial smoking episodes using a three-dimensional profile composed of pleasant, unpleasant and buzz dimensions. Future research should focus upon developing additional items for each of the three dimensions described in this study, replicating the dimensionality in other samples, and creating a standard assessment and scoring strategy to facilitate comparison of results across studies.
